# Cohort Profile: Andhra Pradesh Children and Parents Study (APCAPS)

**DOI:** 10.1093/ije/dyt128

**Published:** 2013-09-07

**Authors:** Sanjay Kinra, KV Radha Krishna, Hannah Kuper, KV Rameshwar Sarma, Poornima Prabhakaran, Vipin Gupta, Gagandeep Kaur Walia, Santhi Bhogadi, Bharati Kulkarni, Aniket Kumar, Aastha Aggarwal, Ruby Gupta, Dorairaj Prabhakaran, K Srinath Reddy, George Davey Smith, Yoav Ben-Shlomo, Shah Ebrahim

**Affiliations:** ^1^Department of Non-Communicable Disease Epidemiology, London School of Hygiene & Tropical Medicine, London, UK, ^2^National Institute of Nutrition, Indian Council for Medical Research, Hyderabad, India, ^3^Public Health Foundation of India, New Delhi, India, ^4^School of Social and Community Medicine, University of Bristol, Bristol, UK, ^5^Department of Anthropology, University of Delhi, Delhi, India, ^6^South Asia Network for Chronic Disease, Public Health Foundation of India, New Delhi, India, ^7^Institute of Health and Biomedical Innovation, Queensland University of Technology, Brisbane, Australia and ^8^Centre for Chronic Disease Control, New Delhi, India

## Abstract

The Andhra Pradesh Children and Parents Study (APCAPS) was originally established to study the long-term effects of early-life undernutrition on risk of cardiovascular disease. Its aims were subsequently expanded to include trans-generational influences of other environmental and genetic factors on chronic diseases in rural India. It builds on the Hyderabad Nutrition Trial (HNT) conducted in 1987–90 to compare the effects on birthweight of a protein-calorie supplement for pregnant women and children. The index children of HNT and their mothers were retraced and examined in 2003–05, and the children re-examined as young adults aged 18–21 years in 2009–10. The cohort was expanded to include both parents and siblings of the index children in a recently completed follow-up conducted in 2010–12 (*N* = ∼6225 out of 10 213 participants). Recruitment of the remaining residents of these 29 villages (*N* = ∼55 000) in Ranga Reddy district of Andhra Pradesh is now under way. Extensive data on socio-demographic, lifestyle, medical, anthropometric, physiological, vascular and body composition measures, DNA, stored plasma, and assays of lipids and inflammatory markers on APCAPS participants are available. Details of how to access these data are available from the corresponding author.

## Why was the cohort set up?

The ‘developmental origins of adult disease hypothesis’ argues that undernutrition in early life plays a critical role in determining an individual’s future risk of cardiovascular disease.^1–4^ The evidence in support of this hypothesis is largely observational but the opportunity to follow up a controlled comparison of a pregnancy and early childhood nutrition intervention in India—the Hyderabad Nutrition Trial (HNT)—was initiated. A long-term follow-up of this trial was conducted from 2003–05 to explore this hypothesis among mothers and children born during the HNT who were still alive and who could be linked to original HNT records.^5^ The follow-up of the birth cohort children as young adults (18–21 years of age) during 2009–10 aimed to study the effect of nutritional shortage in early life on the amount and distribution of body fat and the development of type 2 diabetes and early markers of coronary heart disease. The aim of expanding the cohort in 2010–12, to include parents and siblings of the birth cohort to constitute the Andhra Pradesh Children and Parents (APCAPS) family study, was to explore the trans-generational effects of environmental and genetic risk factors on cardiovascular and other chronic diseases.^6–10^ The main objectives in this phase were: (i) to study whether parental deprivation in childhood (assessed from socio-economic data and height and leg length of the parents) is associated with cardiometabolic risk in the offspring; (ii) to explore differences between maternal and paternal influences on offspring disease risk; (iii) to study whether nutritional supplementation for mothers in pregnancy attenuates this risk; and (iv) to explore differences in cardiometabolic risk factors in children born before and after the nutritional supplementation.

## Who is in the cohort?

### The Hyderabad Nutrition Trial, 1987–90

The Hyderabad Nutrition Trial evaluated the Integrated Child Development Services (ICDS) scheme, a national community outreach programme initiated in 1975 to improve the health, nutrition and development of children in India. Its central focus was the improvement of nutritional status of pregnant and lactating women and children less than 6 years of age, and also included: early childhood education; health, hygiene and nutrition education for the mothers; and delivery of other national programmes (immunisation, anaemia control and basic health care) from the ICDS centres. The programme provided free food in the form of a cereal-based meal prepared from a corn-soya blend and soyabean oil. On average, the meal provided 2.09 MJ and 20–25 g protein to pregnant/lactating women and about 1.25 MJ and 8–10 g protein to children up to 6 years of age. The evaluation was conducted by the National Institute for Nutrition (Hyderabad) during 1987–90, and was funded by the Indian Council for Medical Research and the United States Assistance for International Development. Two adjacent administrative areas, 50–100 km away from Hyderabad city (one with the ICDS programme and the other awaiting implementation at that time), were identified to allow a ‘controlled’ stepped wedge study design involving 29 villages (15 intervention and 14 control villages) of Ranga Reddy district. All women in the reproductive age group of 13–45 years were identified in the initial household enumeration. Women ‘at risk’ of pregnancy were monitored and those who became pregnant were followed closely during pregnancy until childbirth. A clinical examination was carried out at each trimester; attempts were made to weigh the newborns within 48 h of delivery and follow them for the 1st year of life. No published findings exist, but historical records suggest that approximately 4338 births took place. A large amount of data was collected on the pregnant women and ensuing offspring during HNT (socio-demographics; medical and obstetric history for women; feeding and immunisation history for offspring; nutritional supplementation and anthropometrics for both). However, the data collected at different time points were recorded in separate questionnaires which subsequently could not be linked because of a lack of reliable linking identifier and so is unavailable for use. Only birthweight data (which as the primary outcome of the study was recorded in additional files) are available for 603 (40%) of the 1492 children whose identities could be reliably linked to the historical records (see below).

### Long-term follow-up of the Hyderabad Nutrition Trial

As HNT was not planned for long-term follow-up, the records available could not be linked comprehensively to individuals. An ecological approach was used in which children of ages corresponding to 1987–90 births and permanently resident in the villages were recruited and categorized as living in intervention or control villages. This approach allows one to examine what could be achieved in the long term by a nutritional intervention applied to villages from a pragmatic policy perspective.

**Figure 1:**
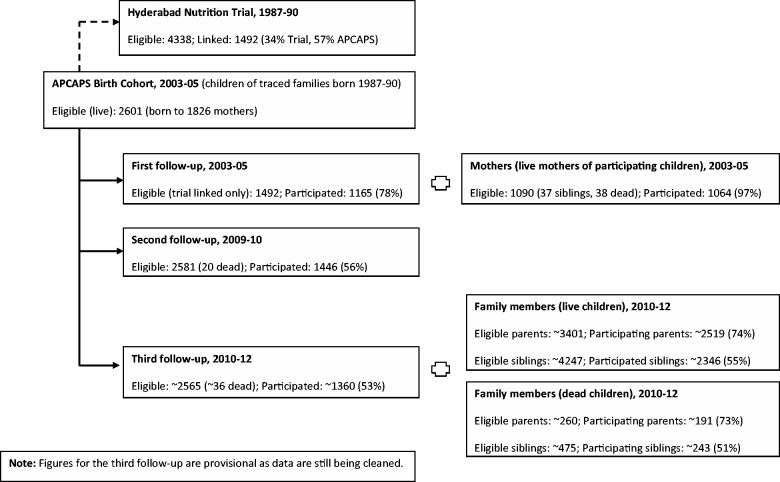
Flow chart of participants in the retrospective and prospective components of Andhra Pradesh Children and Parents Study (APCAPS)

In each village the names of the couples (i.e. parents) available from trial records were used to trace the trial women. A brief socio-demographic questionnaire was completed for women who could be contacted, eliciting information about each child ever borne by her. Local event calendar recall was used to determine the date of birth in many cases. Restricting the households to those with at least one live child born 1987–90 provided the sampling frame for the long-term follow-up study. Of the 2765 names of trial couples available from historical records, 1963 (71%) could be contacted in 2003–05, of whom 1826 had at least one child born during the trial period (1987–90): these constitute the APCAPS trial households. Children born during 1987–90 still alive at the time of the study form the APCAPS birth cohort (*N* = 2601), and these together with the parents and remaining siblings constitute the total membership of the APCAPS family study (*N* = 10 213). A probability algorithm matching on family name pairs (women and their spouses) and child information (date of birth within 6–12 months, birth order, sex and maternal recall of child involvement in the study) was used to retrospectively link the children from the 2003–05 survey to the historical records from the trial. A total of 1492 (57%) children could be reliably linked. Figure 1 summarizes the numbers participating in each phase of the study.

## How often have they been followed up?

### 

#### First follow-up (mothers and children of the APCAPS birth cohort in adolescence, 2003–05)

Only those children who could be reliably linked to their historical records (*N* = 1492) and their mothers were invited to participate in this follow-up. An interviewer-administered questionnaire and clinical examination involving 1165 (78% response; 45% of trial births) adolescent cohort members (intervention group, *N* = 654; control group, *N* = 511) together with a brief questionnaire and examination involving 1064 mothers (out of 1090 mothers alive, 97% response) were carried out at clinics conducted in the study villages.

#### Second follow-up (children of the APCAPS birth cohort in young adulthood, 2009–10)

All 2601 trial children, irrespective of historical linkage, were eligible to participate in a study on DXA measures of body composition and the propensity to develop type 2 diabetes and coronary heart disease. A total of 1446 (56%) out of 2581 children still alive, who were now young adults aged 18–21 years, were examined during this phase.

#### Third follow-up (APCAPS family study, 2010–12)

In 2010, the data collection was extended to the parents and siblings of the trial children, to establish a family cohort study. A total of 6225 (61%) out of a potential of 10 213 individuals participated during this phase (these figures are provisional as data are still being cleaned). In addition, 434 family members from trial families with no living index children were also recruited, yielding data on a provisional total of 6659 participants in this phase.

## Attrition

The flow chart in Figure 1 shows attrition at each stage of the cohort follow-up (for attrition by intervention status, see Appendix Figure 1, available as Supplementary data at *IJE* online). At the time of retracing of the cohort in 2003–05, about a quarter of the families could not be contacted (25% of the intervention group and 33% of the control group). Anecdotal reports from the villagers suggested that the untraced families were likely to have been temporary migrant workers (common at the time of harvest season) who were eligible to participate in the trial. The response rate at the second follow-up (56%) was considerably lower than the first follow-up (78%), possibly due to distance to the clinic. Clinics at the second follow-up were conducted 1–2 h drive away at the National Institute of Nutrition as DXA equipment was required. This differed from the first follow-up, where clinics were held in individual villages. The response rate was particularly low among girls, since many of them had migrated out of the area due to marriage (girls frequently marry before the age of 20 years in this area). Comparison of data collected on households surveyed in 2003–05 suggested that children who took part in the clinics at the first and second follow-ups were more likely to be males and students than those who did not (Table 1). However, there was no difference in birthweights (among the sub-group for whom birthweight data were available). The ∼61% response rate in the most recent follow-up is provisional as the data are still being cleaned. A large proportion of the offspring generation are now in young adulthood and migration is anticipated to be an issue for both men (mostly for work) and women (mostly for marriage). The exact proportion is difficult to predict and may vary depending on local economic opportunities. A positive factor in this is that migration for work and marriage in this area tends to be local (one of the neighbouring villages/towns or Hyderabad city which is 1–2 h drive away).

**Table 1:** Comparison of clinic participants with non-participants at the first (2003–05) and second (2009–10) follow-ups of the Andhra Pradesh Children and Parents Study (APCAPS)^a^

	2003–05			2009–10		
	Participants (*n* = 1165)	Non-participants (*n* = 1436)	*P*-value	Participants (*n* = 1446)	Non-participants (*n* = 1155)	*P*-value
Age (years), mean (SD)^b^	15.9 (0.9)	15.5 (1.2)	<0.001	21.7 (1.1)	21.7 (1.1)	0.09
Female sex, *n* (%)	537 (46.1)	743 (51.7)	0.005	459 (31.7)	822 (71.2)	<0.001
Occupation in 2003–05	(*n* = 1154)	(*n* = 1408)	<0.001	(*n* = 1432)	(*n* = 1130)	<0.001
Full-time student	894 (77.5)	991 (70.4)		1137 (79.4)	743 (65.8)	
Full-time employment	178 (15.4)	310 (22)		214 (14.9)	274 (24.2)	
Other	82 (7.1)	107 (7.6)		76 (5.3)	113 (10)	
Birthweight (g), mean (SD)^c^	2667 (424)	2660 (414)	0.84	2671 (428)	2656 (411)	0.86

SD, standard deviation.

^a^Values are numbers (percentages) unless stated otherwise. *P-*values are based on unpaired *t* tests or *Χ*^2^ tests for heterogeneity with appropriate degrees of freedom.

^b^Mean age estimated at 1 June 2004 (2003–05 survey) and 1 January 2010 (2009–10 survey).

^c^Based on fewer subjects: 2003–05, 544 participants, 228 non-participants; 2009–10, 469 participants, 303 non-participants.

## What has been measured?

The data collected in the three follow-ups are presented in Table 2. In the first follow-up, all data were collected in clinics held at the villages where the participants resided. In the second follow-up, data were collected at a single clinic held at the National Institute of Nutrition. In the third follow-up, data collection was split between the clinics conducted at the National Institute of Nutrition (DXA scans and vascular measures) and in the villages (remaining measures).

**Table 2:** Data collection in Andhra Pradesh Children and Parents Study (APCAPS), 2003–12

Data	First follow-up (2003–05)	Second follow-up (2009–10)	Third follow-up (2010–12)
Participant	Index child (birth linkage), mother^a^	Index child	Index child, parents, siblings
Questionnaire	Socio-demographics Social position (full SLI) Lifestyle e.g. diet, activity (brief questionnaires) General health, medical and family history Atopic history Birth, feeding, immunization history (mother) Household details, socio-demographics (mother)	Socio-demographics Social position (brief SLI) Lifestyle (full questionnaires)^b^ General health, medical and family history Mental health Reproductive health (women)	Socio-demographics Social position (brief SLI) Lifestyle (full questionnaires) General health, medical and family history Mental health Reproductive health (women)
Body composition	Anthropometry (child/mother), skin folds Pubertal status	Anthropometry, skin folds Grip strength DXA scan	Anthropometry, skin folds Grip strength Bio-impedance, DXA scan (subset)
Vascular	Blood pressure Arterial stiffness	Blood pressure Arterial stiffness Carotid intima-media thickness	Blood pressure Arterial stiffness Carotid intima-media thickness
Respiratory	Lung function	Lung function	Lung function
Biological sample	Fasting blood sugar, lipids, insulin DNA (Selected SNPs)	Fasting blood sugar, lipids, insulin Liver, bone function, vitamin D Saliva	Fasting blood sugar, lipids, insulin Liver, bone function, CRP Saliva

SLI: Standard of Living Index scale.

^a^Brief questionnaire and anthropometry only.

^b^Objective measures of physical activity in a subset.

Socio-demographic, health and lifestyle data were collected by questionnaire administered by a trained interviewer. Socio-economic position was assessed using the Standard of Living Index scale, a household level asset-based scale devized for use in Indian surveys.^11^^,^^12^ Data on dietary intake (over the past year) and physical activity (over the past week) were collected by semi-quantitative questionnaires previously adapted and evaluated for use in this setting.^13^^,^^14^ Objective measures of physical activity (accelerometers and actihearts) were also collected on a sub-sample. Anthropometric assessments included weight (measured to the nearest 0.1 kg by digital SECA machine) and height (nearest 1 mm by Leicester plastic stadiometer, Chasmors, UK) and several circumferences (waist, hip, mid-arm, calf and head by a non-stretch metallic tape). Skin folds were measured at five sites (biceps, triceps, subscapular, suprailiac and calf) to the nearest 0.2 mm using the Holtain caliper. Grip strength was measured by a hand-held dynamometer (Grip D, Takei, Japan in the second follow-up, and Lafayette 78010 in the third follow-up), separately for each arm. Body composition was assessed in the second follow-up by whole-body scan performed by DXA (Hologic models Discovery A or 4500W), after excluding pregnant women. Standard Hologic software options were used to define regions of the body. In the third follow-up, body composition was assessed in the village clinics using portable TANITA digital body composition analyser (TANITA BC 418 M57NA) which records the total and segmental body composition measurements, whereas DXA scans were done in a subset of subjects at the National Institute of Nutrition. Blood pressure was measured at the right arm in the sitting position using an oscillometric device (Omron M5-I). Arterial stiffness (augmentation index and pulse wave velocity) was measured in the supine position using the Sphygmocor (Atcor Medical) in the first and second follow-ups, and Vicorder (Skidmore Medical UK) in the second and third follow-ups, respectively. Lung function (forced vital capacity and expiratory volume) was measured in the second follow-up by MicroPlus spirometer (Micromedical), after three acceptable blows from a maximum of eight blows; Card-Guard Spiro-Pro was used for lung function assessment in the third follow-up. Carotid intima media thickness was measured at the near and far carotid area close to the bulb by a B-mode ultrasound scanner (Ethiroli Tiny-16a, Surabhi Biomedical Instrumentation, India) and analysed using semi-automated software. Fasting blood samples were collected and separated for storage, and assays for haemoglobin, glucose, cholesterols (HDL, total), triglycerides and insulin (by ELISA) have been performed at each follow-up; in addition, liver and bone functions have been assayed in second and third follow-ups, and the C-reactive protein (Hs) in the third follow-up only. Demographic and socio-economic data were collected from the official village records in the local government (Panchayat) offices in 2003–05 and are currently being compared with data on village development available from census records over the period 1981–2001 (2011 awaited) to track the development of the villages.

## What has it found? Key findings and publications

No publications resulted from the initial trial except an abstract in an internal publication based on interim analyses which suggested an 88g higher birthweight in children born in the intervention villages. A later reanalysis of available birthweights from the trial data suggested a more modest 61g (95% CI 18–104, *P* = 0.007) higher birthweight in the intervention village children.^5^ The details of the trial methodology and the subsequent attempt at tracing and establishment of the birth cohort were published in the primary manuscript from the first follow-up,^5^ and more comprehensively in a PhD thesis (S.K. Kinra, University of Bristol 2007, unpublished). The data from the first follow-up were consistent with the developmental origins of adult disease hypothesis, suggesting a lower risk of cardiovascular disease (as evidenced by arterial stiffness and insulin resistance) in adolescents from the intervention villages, as compared with controls (see Table 3).^5^ They were also 14 mm taller, but not any more obese compared with controls. In another analysis, relative leg length was not found to be associated with nutritional supplementation, challenging the use of leg length as a biomarker for early childhood nutrition, which has been based mainly on observational data.^15^ Other publications from this cohort in the adolescent phase have examined: (i) the social patterning of cardiometabolic risk (no clear associations between socioeconomic position and cardiometabolic risk factors except adiposity which had a positive association with socioeconomic position in adolescence); (ii) the value of different anthropometric measures in predicting cardiometabolic risk (associations between several anthropometric measures and cardiometabolic risk, but no clear discrimination between them); and (iii) the role of intergenerational undernutrition in conferring increased cardiometabolic risk (no association between maternal leg length and offspring cardiometabolic risk except adiposity)(manuscripts submitted). Data from the recently completed follow-up in young adulthood are currently being analysed and several manuscripts are under various stages of peer review: early findings suggest a lack of long-term effect of nutritional supplementation on body composition and lung function in young adulthood, which were more influenced by contemporary lifestyle factors. Preliminary analysis of data for 20 villages from the family study suggests a high prevalence of chronic diseases and their risk factors in adults aged 30–84 years, such as: hypertension (BP > 140/90 mmHg: men 20%, women 13%); overweight (BMI ≥ 25 kg/m^2^: men 18%, women 24%); underweight (BMI < 18.5 kg/m^2^: men 31%, women 20%); and smoking (men 50%, women 1%). With the current rising trends in chronic diseases in low- and middle-income countries, the results from this family cohort study in a largely rural setting should provide crucial insights into the potential reasons for these trends.

**Table 3:** Association between supplemental nutrition and cardiovascular risk in adolescence: First follow-up of the Andhra Pradesh Children and Parents Study (APCAPS), 2003–05

Risk factor	Mean (SD)		Mean difference (95% CI): control minus intervention	
	Intervention (*n* = 633)	Control (*n* = 498)	Unadjusted	Adjusted^a^
% female	47	45		
Age (years)	15.8 (0.9)	15.9 (0.9)	NA	NA
Height (mm)	1159 (83)	1549 (82)	−10.0 (−18.7 to −1.4)	−13.6 (−23.1 to −4.1)
Fat mass index (kg/m^2^)	2.6 (1.3)	2.6 (1.5)	−0.02 (−0.23 to 0.19)	0.04 (−0.10 to 0.18)
Systolic blood pressure (mmHg)	108.7 (10.3)	109.6 (10.0)	0.83 (−1.44 to 3.11)	0.59 (−1.11 to 2.29)
Augmentation index (%)	2.5 (11.4)	5.6 (9.4)	3.16 (0.8 to 5.51)	3.30 (0.96 to 5.65)
Total cholesterol (mmol/l)	3.45 (0.69)	3.45 (0.67)	0 (−0.12 to 0.12)	−0.02 (−0.11 to 0.08)
HOMA score^b^	3.16 (1 to 10)	3.79 (1.2 to 11.7)	0.18 (0.04 to 0.32)	0.18 (0.03 to 0.33)

^a^Adjusted for age, sex, pubertal stage, standard of living index, village population, room temperature (blood pressure) and heart rate (augmentation index).

^b^HOMA (homeostasis model assessment) score is on log scale (geometric mean/95% reference range presented); 0.18 mean difference equates to 20% (95% CI: 3 to 39%) on the original scale.

## What are the main strengths and weaknesses?

The Andhra Pradesh Children and Parents Study is a unique family study in a transitioning rural population, that can provide valuable information on the underlying patterns in risk factors for cardiometabolic disorders. The inbuilt nutritional intervention component provides additional opportunities to evaluate whether such programmes in an undernourished population have long-term implications for the health of subsequent generations. The original trial with a controlled design should reduce, but does not fully overcome, chances of bias and confounding. The detailed phenotypic data and the collection of blood or saliva samples from entire families for genetic studies provide a major intergenerational resource for future research and should delineate the genetic and environmental determinants and their contribution to a host of outcomes in this population.

The main limitation of this cohort is the lack of follow-up data during infancy and childhood (some follow-up over infancy was conducted but data linkage is limited) and hence a loss of opportunity for comparison of postnatal and childhood growth patterns between the two groups. We do not have prospectively collected data on infant and childhood morbidity and mortality which could have provided valuable clues to the impact of the nutritional supplementation programme on these indices. However, our analysis of retrospectively collected data (using birth/death data on all children ever born to the women, collected at the time of initial tracing survey of 2003–05) suggests some effects of nutrition on these indices. The 1963 traced women had delivered a total of 8246 children (4.2 children per woman), of whom 952 had died. The estimated child death rates were 10% for intervention and 13% for control areas; the broad consistency of the child death rates with Indian national figures over that duration (∼11.5% under-five mortality in 1990) provides some reassurance on the completeness of the initial tracing exercise.^16^ The loss to follow-up was substantial, therefore selection bias cannot be ruled out. However, baseline comparisons of available socio-demographic data on participating and non-participating children did not suggest any major differences (Table 1). The response rate also compares favourably with other similar studies involving long-term follow-up of nutrition trials.^1^,^4^

The analyses by the original trial design are limited by a lack of direct evidence on who took the nutritional supplement (primary analyses are ecological, based on the area and time of birth) and the amount of supplement consumed. In the trial, considerable efforts were made to ensure uptake of the supplement, including direct observation. This coupled with a higher birthweight of children born in the intervention area, comparable in magnitude to other trials of nutritional supplementation, suggests that the intervention was effective. Although the ecological design could lead to an underestimation of the efficacy of nutritional supplement, it provides a more pragmatic estimate of the effectiveness of such nutritional supplement programmes in real-world situations, which has important policy implications. Bias could also have arisen from differing rates of urbanization in the intervention and control groups. The study area has over the past 10 years shown definite trends in urbanization, with a large number of educational institutes (schools, colleges and vocational/industrial training centres) mushrooming in the area. The state government also built a new international airport in the vicinity, buying up agricultural land and contributing to a sudden increase in the socio-economic status of many nearby villages and their inhabitants. Although this may be a limitation for trial-based analyses, the natural experiment arising from the rapid but uneven economic development of the area also provides unique opportunities to investigate the mechanisms by which socio-economic development and urbanization increase the risk of chronic diseases.

## Can I get access to the data? Where can I find more?

The investigators of APCAPS welcome collaborations, which are considered by the steering committee through a standard procedure involving review of a brief proposal. To discuss, please contact Sanjay Kinra (Sanjay.Kinra@lshtm.ac.uk).

## Supplementary Data

Supplementary data are available at *IJE* online.

## Funding

Hyderabad Nutrition Trial (United States Assistance for International Development and the Indian Council for Medical Research to R.S.); first follow-up (Royal College of Physicians Eden Fellowship in Paediatrics 2002 to S.K.); second follow-up (Wellcome Trust Grant 083707 to H.K.); third follow-up (Wellcome Trust Strategic Award 084774 to S.E. and European Commission Strategic Award (FP-7) to S.E. and S.K.). The National Institute of Nutrition (Directors), Indian Council for Medical Research, provided support in kind to each of the three follow-ups through free or subsidized access to facilities and materials.

PhD studentships: second follow-up (Amy Taylor, Wellcome Trust Award to the University of Bristol; Ruth Sullivan, Economic & Social Research Council UK Award to the London School of Hygiene & Tropical Medicine; B.K., Queensland University of Technology, Australia); third follow-up (P.P., Wellcome Trust Capacity Strengthening Strategic Award to the Public Health Foundation of India and a consortium of UK universities, grant 084754).

## Supplementary Material

Supplementary Data
