# Supplementary material for: Cohort Profile: Andhra Pradesh Children and Parents Study (APCAPS)
Source: Int J Epidemiol. 2013 Sep 7;43(5):1417–24. doi: 10.1093/ije/dyt128 (PMC4190511; doi:10.1093/ije/dyt128)
Supplement: Supplementary Data [file supp_43_5_1417__index.html]

Supplementary Data 

# Cohort Profile: Andhra Pradesh Children and Parents Study (APCAPS)

## Supplementary Data

files

**Files in this Data Supplement:**

- Supplementary Data - pdf file
