# Supplementary material for: Cohort Profile: Andhra Pradesh Children and Parents Study (APCAPS)
Source: Int J Epidemiol. 2013 Sep 7;43(5):1417–24. doi: 10.1093/ije/dyt128 (PMC4190511; doi:10.1093/ije/dyt128)
Supplement: Supplementary Data [file supp_dyt128_Supplementary_data.pdf]

Appendix Figure 2: Attrition by intervention status in Andhra Pradesh Children and Parents Study (APCAPS)

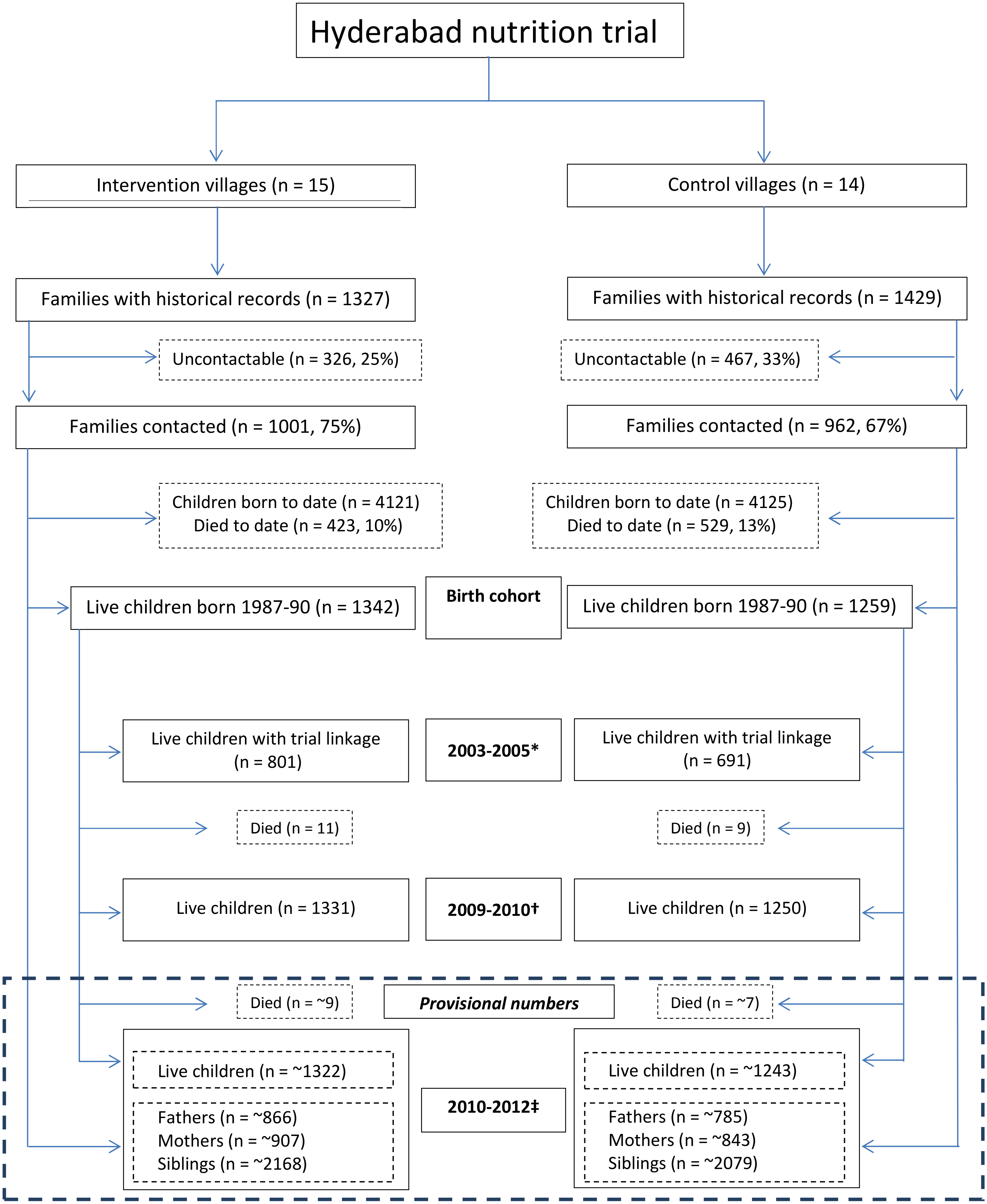

Notes
* These children were born to 1441 mothers: intervention (n = 766), control (n = 675)
† These include those who migrated out of villages: intervention (n = 326), control (n = 366)
‡ These provisional numbers (data are still being cleaned) are for family members of the live children. In addition, family members of some trial families with no current live children from the trial were also recruited in this follow-up. The provisional numbers including these families are as follows:

Intervention: index children (n =~ 1322), fathers (n = ~910), mothers (n = ~971), siblings (n =~2369)

Control: index children (n =~ 1243), fathers (n = ~853), mothers (n = ~927), siblings (n = ~2353)
